# Supplementary material for: Efficacy of AAV8-hUGT1A1 with Rapamycin in neonatal, suckling, and juvenile rats to model treatment in pediatric CNs patients
Source: Mol Ther Methods Clin Dev. 2020 Dec 3;20:287–97. doi: 10.1016/j.omtm.2020.11.016 (PMC7809245; doi:10.1016/j.omtm.2020.11.016)
Supplement: Document S1. Figures S1–S9 and Table S1 [file mmc1.pdf]

OMTM, Volume *20*

Supplemental Information

Efficacy of AAV8-h*UGT1A1* with Rapamycin in neonatal, suckling, and juvenile rats to model treatment in pediatric CNs patients

Xiaoxia Shi, Sem J. Aronson, Lysbeth ten Bloemendaal, Suzanne Duijst, Robert S. Bakker, Dirk R. de Waart, Giulia Bortolussi, Fanny Collaud, Ronald P. Oude Elferink, Andrés F. Muro, Federico Mingozzi, Giuseppe Ronzitti, and Piter J. Bosma

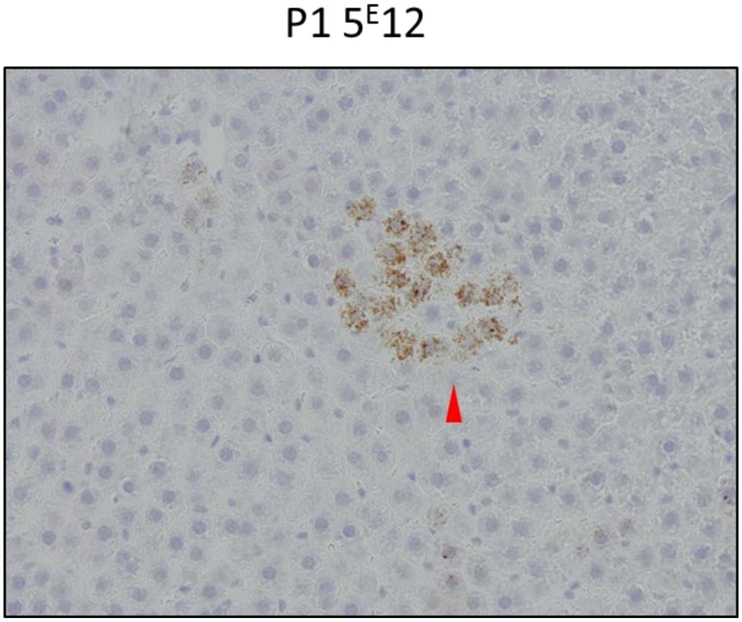

Supplemental Figure 1. Cluster of h*UGT1A* mRNA expressing hepatocytes at 12 weeks after injection of 5x10^{12} vg/kg in 1 day old rats.
RNA Scope images (20x) visualizing h*UGT1A* mRNA expression in liver tissue.

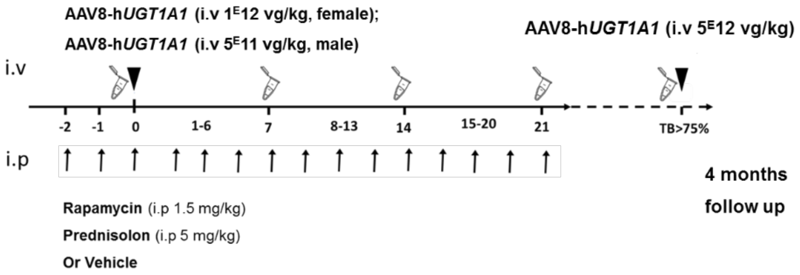

Supplemental Figure 2. Rapamycin experiment setup.
12 days old Gunn rats received daily immune suppressive regimen (IS regimen) by i.p. administration of vehicle, rapamycin with or without prednisolone. Two days later, a suboptimal dose of AAV8-h*UGT1A1* was administered by tail vein injection. The IS regimen continued up to 21 days after vector administration. Upon loss of correction, serum total bilirubin level > 75% of untreated rats, AAV8-*hUGT1A1* (5x10e12 vg/kg) was re-administered in combination with the same IS regimen. Blood was sampled by tail vein puncture to monitor serum total bilirubin, and to determine (neutralizing) antibodies towards AAV8 capsid.

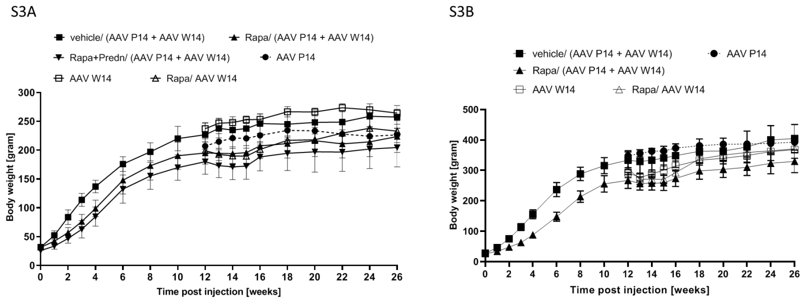

Supplemental Figure 3. Rapamycin with or without prednisolone delay the growth of rats.
The body weight of female (A) and male (B) rats were measured every 1 or 2 weeks after AAV administration. Rats in each group n=3-5. Data are shown as mean ± SD.

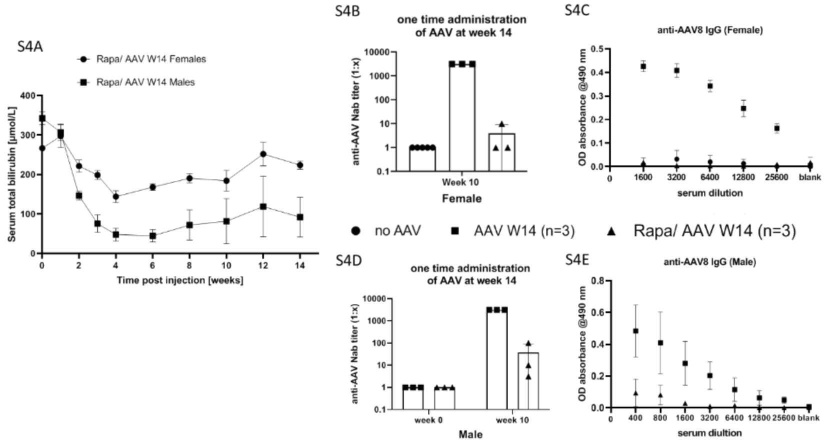

Supplemental Figure 4. Rapamycin prevents the humoral immune response against AAV8 in 14 week old rats, but does not increase liver transduction efficiency.
14 weeks old naïve rats received i.v. administration of 5 x 1012 vg/kg AAV8-hUGT1A1 (n=3) in combination with i.p. administration of daily i.p. injections of rapamycin, starting 2 days before vector until 21 days after vector administration. Blood was sampled by tail vein puncture every 1 or 2 weeks to monitor serum total bilirubin (A). At 10 weeks after AAV administration the level of anti-AAV8 IgG and neutralizing antibodies in serum was determined. Anti-AAV8 neutralizing antibodies (NAbs) in female (B) and male (D). Anti-AAV8 IgG levels in female (C), male (E) rats. Data are shown as mean ± SD.

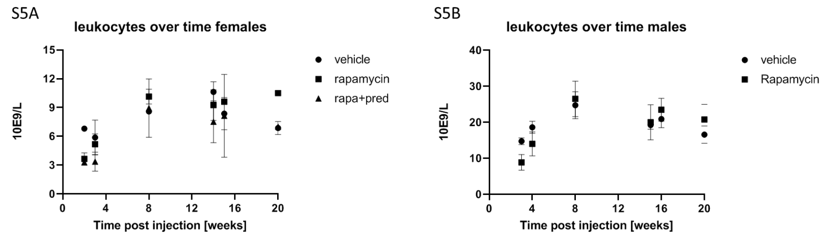

Supplemental Figure 5. Rapamycin and prednisolone result in mild leukopenia in rats.
The leukocytes levels in rats receiving i.p administration of vehicle, rapamycin with or without prednisolone at week 1-3 and week 12-15 were determined at the after the last i.p. injection. (A) and male (B). Rats in each group n=3-5. Data are shown as mean ± SD.

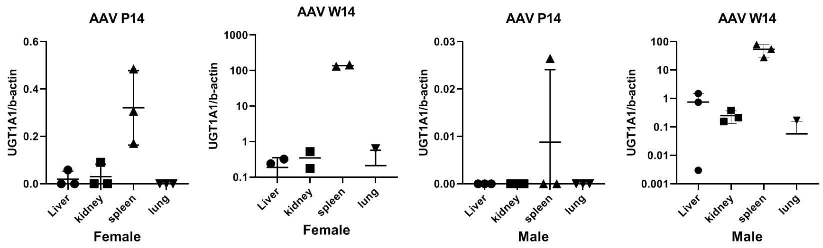

Supplemental Figure 6. Increased presence of AAV vector genomes in the spleen in older animals.
The vector genomic copies in tissues of rats that were naïve at the time of AAV administration that were injected only once, at P14 or W14, and did not receive any immune suppressive. Data are shown as mean ± SD.

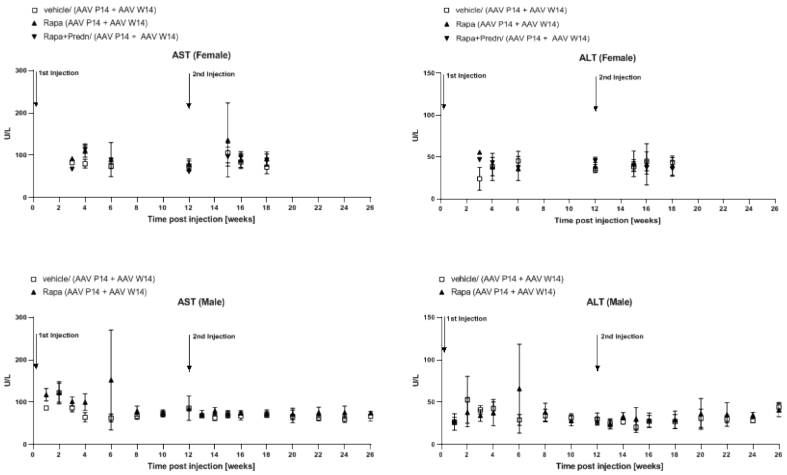

Supplemental Figure 7. Levels of serum AST and ALT overtime.
Blood was sampled by tail vein puncture every 1-2 weeks after AAV8-h*UGT1A1* administration and aspartate aminotransferase (AST) and alanine aminotransferase (ALT) in plasma were determined. The data presented are the mean ± SD at different time points for all groups.

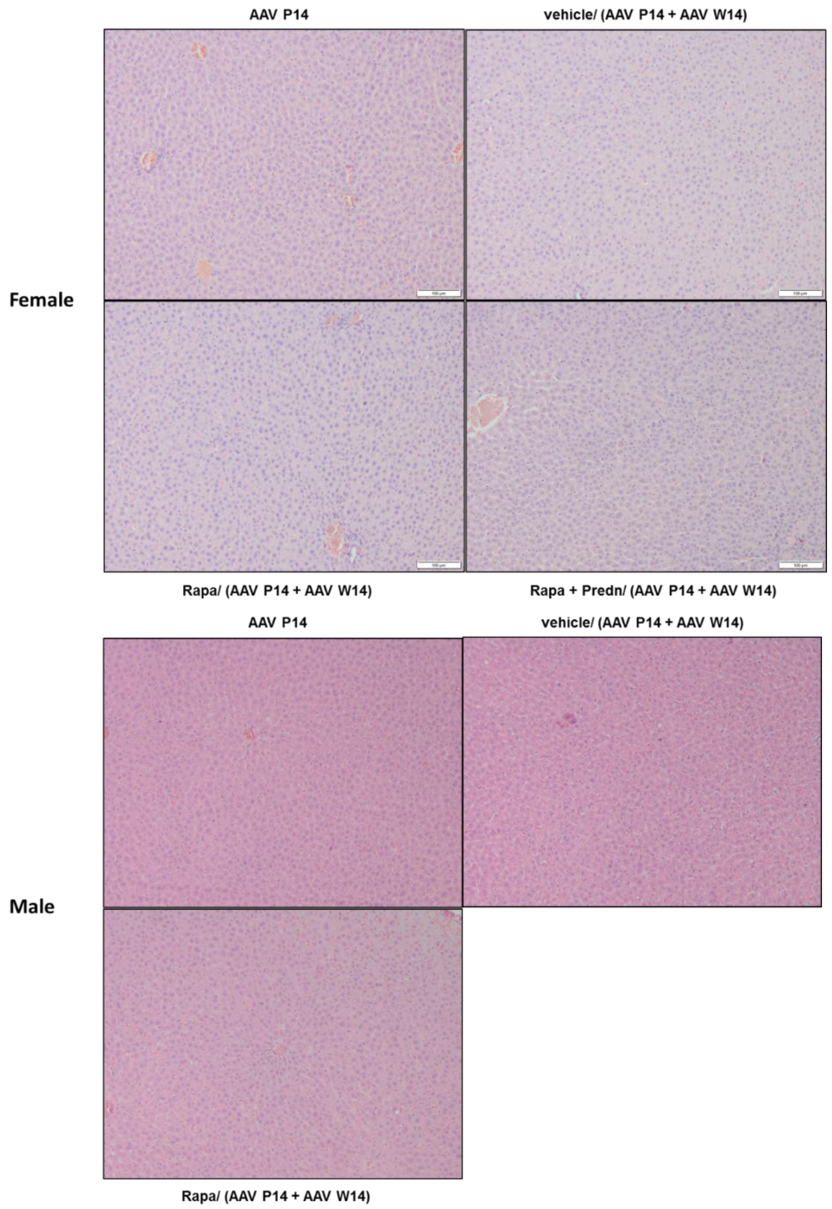

Supplemental Figure 8. Representative images of liver HE staining of rats (20x) at the time of sacrifice.

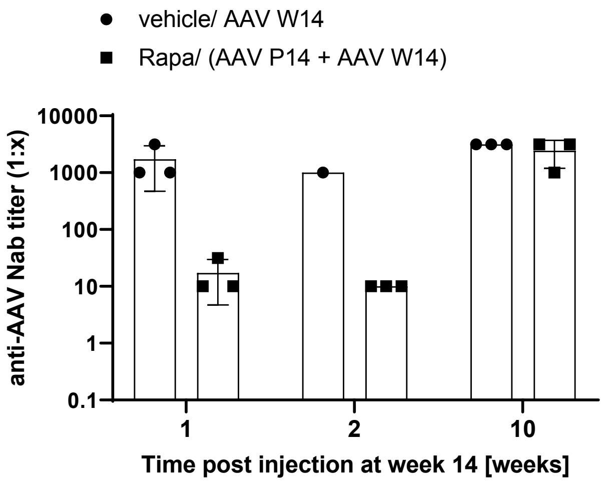

Supplemental Figure 9. Rapamycin treatment delays but does not prevent AAV NAbs formation after the second administration of vector.
Previously injected female rats, received daily i.p. administration of rapamycin starting 2 days before (t = -2) until 21 days (t=21) after re-administration of AAV8-h*UGT1A1* in 14 weeks old animals. 14 weeks old naïve rats received the same dose of AAV8-h*UGT1A1*. At 1, and 2 weeks and 10 weeks after vector administration the level of neutralizing antibodies in serum was determined. Data represent the mean ± SD.

Supplemental Table 1.

Oligonucleotide qRT-PCR primers used for vector detection of *hUGT1A1* in gDNA and cDNA isolated from liver, as well as probes for RNA *in situ* hybridization on liver tissue. And the reference gene *β-actin*.

Target	Sense	Antisense
h*UGT1A1*	5′-GGCGGGCGACTCAGATC-3′	5′-GGGAGGCTGCTGGTGAATATT-3′
β-actin	5′-AGCCATGTACGTAGCCATCCA-3′	5′-TCTCCGGAGTCCATCACAATG-3′
