# Supplementary material for: Efficacy of AAV8-hUGT1A1 with Rapamycin in neonatal, suckling, and juvenile rats to model treatment in pediatric CNs patients
Source: Mol Ther Methods Clin Dev. 2020 Dec 3;20:287–97. doi: 10.1016/j.omtm.2020.11.016 (PMC7809245; doi:10.1016/j.omtm.2020.11.016)
Supplement: Document S2. Article plus supplemental information [file mmc2.pdf]

Molecular Therapy
Methods & Clinical Development
Original Article

Efficacy of AAV8-h*UGT1A1* with Rapamycin in neonatal, suckling, and juvenile rats to model treatment in pediatric CNs patients

Xiaoxia Shi,[1] Sem J. Aronson,[1] Lysbeth ten Bloemendaal,[1] Suzanne Duijst,[1] Robert S. Bakker,[1] Dirk R. de Waart,[1] Giulia Bortolussi,[2] Fanny Collaud,[3,4] Ronald P. Oude Elferink,[1] Andrés F. Muro,[2] Federico Mingozzi,[3] Giuseppe Ronzitti,[3,4] and Piter J. Bosma[1]

[1]Amsterdam UMC, University of Amsterdam, Tytgat Institute for Liver and Intestinal Research, AGEM, Meibergdreef 69-71, 1105 BK Amsterdam, the Netherlands; [2]International Centre for Genetic Engineering and Biotechnology, 34149 Trieste, Italy; [3]Genethon, 91000 Evry, France; [4]Université Paris-Saclay, Université d'Evry, INSERM, Genethon, Integrare Research Unit UMR S951, 91000 Evry, France

A clinical trial using adeno-associated virus serotype 8 (AAV8)-human uridine diphosphate glucuronosyltransferase 1A1 (h*UGT1A1*) to treat inherited severe unconjugated hyperbilirubinemia (Crigler-Najjar syndrome) is ongoing, but preclinical data suggest that long-term efficacy in children is impaired due to loss of transgene expression upon hepatocyte proliferation in a growing liver. This study aims to determine at what age long-term efficacy can be obtained in the relevant animal model and whether immune modulation allows re-treatment using the same AAV vector. Neonatal, suckling, and juvenile Ugt1a1-deficient rats received a clinically relevant dose of AAV8-h*UGT1A1*, and serum bilirubin levels and anti-AAV8 neutralizing antibodies (NAbs) in serum were monitored. The possibility of preventing the immune response toward the vector was investigated using a rapamycin-based regimen with daily intraperitoneal (i.p.) injections starting 2 days before and ending 21 days after vector administration. In rats treated at postnatal day 1 (P1) or P14, the correction was (partially) lost after 12 weeks, whereas the correction was stable in rats injected at P28. Combining initial vector administration with the immune-suppressive regimen prevented induction of NAbs in female rats, allowing at least partially effective re-administration. Induction of NAbs upon re-injection could not be prevented, suggesting that this strategy will be ineffective in patients with low levels of preexisting anti-AAV NAbs.

INTRODUCTION

Crigler-Najjar syndrome (CNs) type 1, the most severe form of inherited unconjugated hyperbilirubinemia, is lethal during infancy or early childhood.[1] This autosomal-recessive liver disorder is caused by the deficiency of a hepatic enzyme, uridine diphosphate glucuronosyltransferase 1A1 (UGT1A1).[2] UGT1A1 converts the toxic and lipophilic unconjugated bilirubin (UCB) to water-soluble conjugated bilirubin (CB) that can be excreted into bile by active transport across the hepatocyte canalicular membrane.[2,3] Deficiency of UGT1A1 leads to UCB accumulation in all tissues, causing irreversible and lethal brain damage, characterized by kernicterus, the yellow pigmentation of the globus pallidus in the basal ganglia. Current treatments consist of intensive phototherapy, a cumbersome treatment that becomes less effective over time.[4,5] For the most severely affected patients, a liver transplantation is inevitable at some point in their lifetime.[4–6] Important shortcomings of liver transplantation, such as donor availability, procedure-associated complications and mortality, graft survival, and increased cancer and infection risks due to life-long need for immune suppression, indicate that alternative treatments are urgently needed.[7]

A promising alternative curative treatment for inherited severe liver disorders, such as CNs, is *in vivo* gene therapy using recombinant adeno-associated virus (AAV) vectors. Liver-directed gene therapy trials for hemophilia B, a bleeding disorder caused by factor IX deficiency, have achieved a sustained reduction of bleeding episodes after a single systemic injection of an AAV vector containing cDNA encoding the human factor IX protein.[8,9] The safety and efficacy of this treatment strategy is currently being investigated for several monogenic inherited severe liver disorders. Clinical trials are ongoing for ornithine transcarbamylase deficiency (ClinicalTrials.gov: NCT02991144), familial hypercholesterolemia (ClinicalTrials.gov: NCT02651675), and glycogen storage disease type Iα (ClinicalTrials.gov: NCT03517085), and also the feasibility of liver-directed gene therapy to treat severe CNs is currently being investigated in clinical trials (ClinicalTrials.gov: NCT03466463 and NCT03223194).

Recombinant AAV vectors do not actively integrate into the host genome and are lost upon cell division.[10] Studies in neonatal animals, modeling AAV gene therapy to treat CNs early after birth, showed loss of correction over time due to hyper-proliferation of hepatocytes in a

Received 11 September 2020; accepted 28 November 2020;
https://doi.org/10.1016/j.omtm.2020.11.016.

Correspondence: Piter J. Bosma, Amsterdam UMC, University of Amsterdam, Tytgat Institute for Liver and Intestinal Research, AGEM, Meibergdreef 69-71, 1105 BK Amsterdam, the Netherlands.
E-mail: p.j.bosma@amsterdamumc.nl

This is an open access article under the CC BY license (http://creativecommons.org/licenses/by/4.0/).

growing liver.[11,12] Although in CNs phototherapy can prevent brain damage, the treatment is cumbersome, losses efficacy associated with patient growth, and severely affected patients remain at risk to develop irreversible brain damage due to sudden spikes of UCB. A world CNs registry indicated that a significant percentage of the severely affected patients die during early childhood when access to adequate treatment is limited, but early diagnosis and dedicated therapy results in a near normal to normal life expectancy (S.J. Aronson; F. Mingozzi; P.J.Bosma, unpublished data). Treating severely affected patients early after birth will prevent lethal brain damage during childhood. An additional argument to aim at gene therapy early in life is that, due to the exposure to wild-type AAV, the prevalence of neutralizing antibodies (NAbs) toward AAV serotype 8 (AAV8) in the population increases with aging.[13,14] A screening of adult CNs patients revealed that 30.6% had detectable levels of NAbs toward AAV8.[15]

To determine at what age gene therapy would result in long-term efficacy when using the vector that is also evaluated in an ongoing clinical trial, Ugt1a1 deficient rats received a clinically relevant dose either at neonatal or juvenile age. In case of loss of correction over time, re-treatment will be necessary to retain therapeutic efficacy. The high titer of NAbs toward the vector induced by the first administration will impair hepatocyte transduction efficiency of a second administration unless we are able to reduce the initial formation of NAbs. Immune suppression to reduce vector capsid-mediated B and T cell activation could prevent or reduce NAb formation, and, if effective, would render initial treatment earlier after birth and re-treatment after liver maturation feasible. The efficacy of an immune-suppressive regimen of rapamycin, based on shifting the T cells toward an increased presence of regulatory T cells,[16] was studied in sucking Ugt1a1 deficient rats to model its suitability in children suffering from CNs type 1. An effective strategy to block NAbs toward AAV8 has relevance beyond application in young patients, since it will also allow re-treatment of patients who may receive a sub-optimal dose as part of the clinical trial. Furthermore, liver damage due to, for instance, a viral infection or use of alcohol may result in loss of correction, rendering a re-treatment needed later in life.

RESULTS

Long-term efficacy of AAV8-h*UGT1A1* depends on the age of treatment

To model long-term efficacy in children of a clinically relevant dose of AAV8-h*UGT1A1,* the vector similar to the one used in the ongoing clinical trial for CNs, patients (CureCN), Ugt1a1 deficient rats 1, 14, and 28 days of age received 5×10^{12} vector genomes (vg)/kg by intravenous injection (Figure 1A). To monitor the effect on serum bilirubin, blood was sampled every 2 or 4 weeks after vector administration. Independent of age and sex, treatment with AAV8-h*UGT1A1* resulted in complete normalization of total serum bilirubin levels at 2 weeks after injection. In rats treated at 28 days after birth, this complete normalization persisted in males, while in females after 12 weeks a low level of serum bilirubin was detectable (Figure 1B). Treatment at postnatal day 1 (P1) or P14 resulted in complete correction followed by a gradual increase of serum bilirubin levels over time (Figures 1C and 1D). After 12 weeks serum bilirubin levels in rats treated at P1 reached that of untreated controls. This loss of correction seems due to the reduced presence of vector genomes in hepatocytes, although the observed decrease only reached statistical significance between injections at P1 and P28 (Figure 1E). The reduced presence of hepatocytes expressing *UGT1A1* mRNA is also in agreement with the loss of correction (Figure 1F). In most liver slides of rats injected at P1, no *UGT1A1* mRNA-positive hepatocytes were detected, but a cluster of positive cells was seen in some, most likely resulting from proliferation of a hepatocyte in which the vector has integrated into the host genome (Figure S1). The rapid loss over time of UGT1A1-expressing hepatocytes and the loss of expression confirms the data previously reported for the Ugt1 knockout (KO) mouse model.[11] The age at treatment also affected the immune response toward the AAV vector. Only 5 out of 10 rats injected at P1 had a detectable titer of anti-AAV antibodies while all rats injected at P14 already had high titers against the vector at 8 weeks after injection (data not shown).

Rapamycin blocks humoral response toward AAV8 capsid

The loss of therapeutic efficacy when treating suckling rats implies that AAV vector re-administration is needed to obtain life-long correction when aiming to treat these young animals. The presence of a high titer of antibodies toward the vector induced by the first treatment blocks hepatocyte transduction, rendering re-administration ineffective.[17] We hypothesized that immune suppression covering the exposure of the vector to the immune system using rapamycin and prednisolone may prevent this. In addition, rapamycin seems a promising option because it increases autophagy, resulting in an increased liver transduction in non-human primates (NHPs).[18] Considering the heterogeneous anti-AAV response in rats treated at P1, the rats treated at P14 were used to study the efficacy of rapamycin, with or without the addition of prednisolone, previously shown to prevent immune responses toward hepatocytes transduced with AAV.[9] Deliberately inducing a transient correction by using a sub-optimal first dose of AAV8-h*UGT1A1* (1×10^{12} vg/kg) allows demonstration of the efficacy of a second vector administration. Immune suppression was given for 3 weeks by daily intraperitoneal (i.p.) administration of rapamycin, with or without prednisolone, or vehicle, starting 2 days before vector administration, until 21 days after vector administration (Figure S2). This regimen completely prevented the formation of anti-AAV8 immunoglobulin G (IgG) in female rats (Figure 2A). To determine the presence of NAbs, a reporter vector AAV8-luciferase was preincubated with increasing dilutions of sera from treated or control animals. Subsequently, the neutralizing effect was determined by comparing the transduction efficacy in HEK293T cells.[19] The neutralizing titer of the sample was determined as the highest dilution at which 50% or greater inhibition of the luciferase expression was measured. As shown in Figure 2B, in rapamycin-treated rats the titer was similar to that in serum from naive female rats, irrespective of the presence of prednisolone. The NAb titer in vehicle-treated rats was very high; only upon diluting the serum 1,000-fold did luciferase expression in HEK293T cells reach 50% of that of serum-free medium (Figure 2B). In rodents, AAV8 liver transduction efficiency is higher in males.[20,21] To ensure

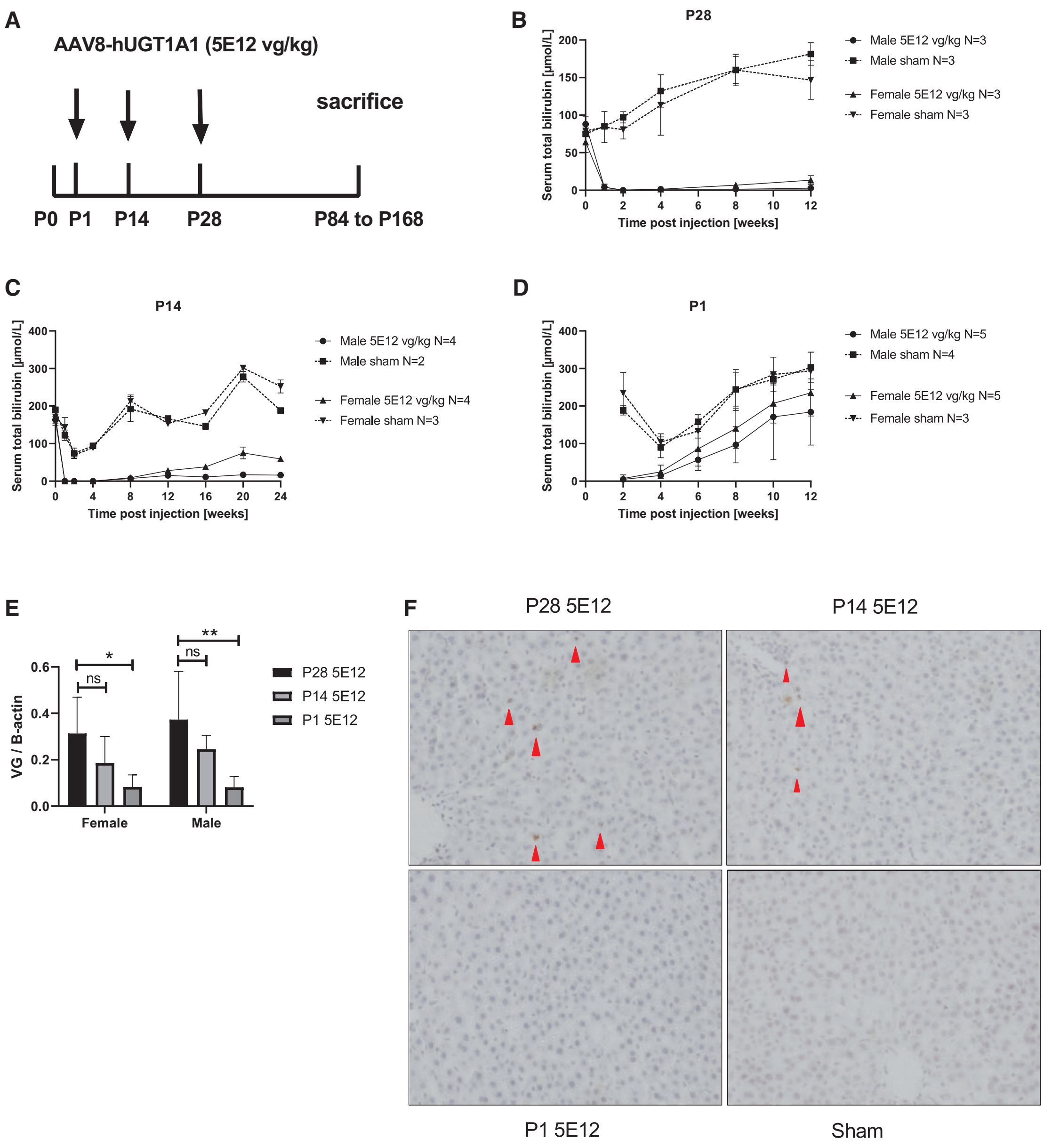

Figure 1. Age at treatment affects long-term correction and immune response toward AAV vector

(A) Experimental setup. (B–D) Serum bilirubin levels over time in rats treated at days 1, 14, or 28 after birth with 5×10^{12} vg/kg of AAV8-h*UGT1A1* or sham. (E) The vector genomic copies in the liver at the time of sacrifice. (F) Representative RNA Scope images (×20) visualizing h*UGT1A* mRNA expression in male liver tissue. Data represent mean ± SD. Statistical analyses were performed by two-way ANOVA.

a transient correction pattern in male rats, a 2-fold lower dose of AAV8-h*UGT1A1* was used (5×10^{11} vg/kg), and because no additive effect of prednisolone was seen in females, the combination of rapamycin and prednisolone was omitted. This dose did not induce a detectable titer of anti-AAV8 IgG but did induce NAbs toward AAV8, albeit with a low titer not reaching a statistically significant difference with the serum from naive rats (Figures 2C and 2D). This reduced response could be due to the 50% lower dose or a less mature immune system in males or a combination of both.

Absence of NAbs toward AAV8 allows effective re-treatment with AAV8-h*UGT1A1* but is transient in adult female rats

Administration of 1×10^{12} vg/kg AAV8-h*UGT1A1* at P14 in female rats resulted in a transient correction, with the serum bilirubin level returning to that of untreated rats at 12 weeks after vector injection (Figure 3A). Rapamycin with or without prednisolone resulted in some delay in the growth of juvenile rats that did not reach statistical significance (Figure S3). After having shown that rapamycin blocked the formation of antibodies directed against the AAV8 vector

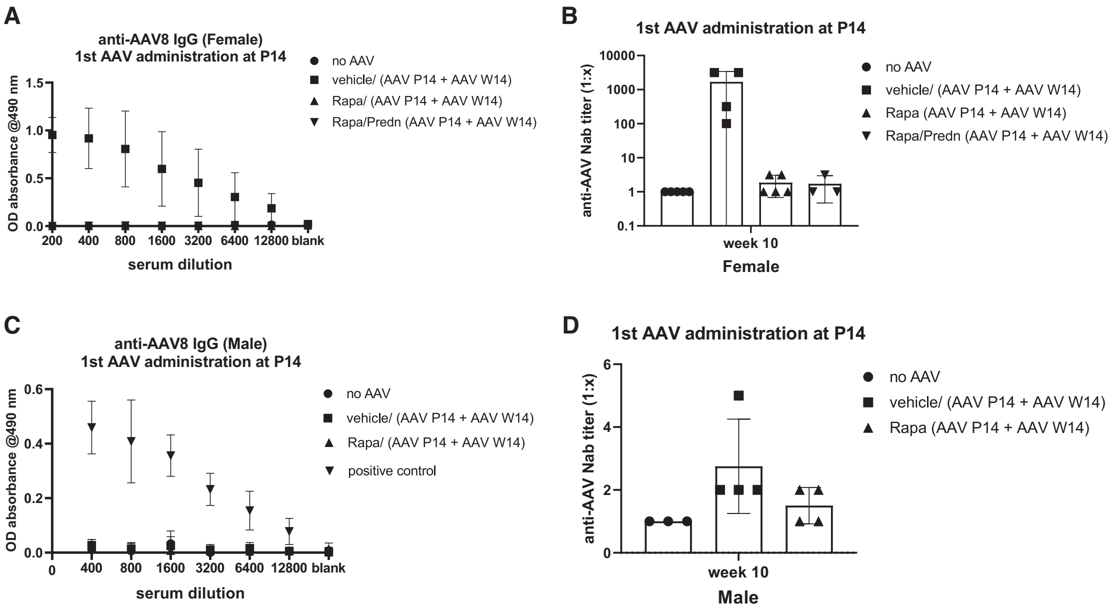

Figure 2. Rapamycin treatment blocks anti-AAV IgG and neutralizing antibody (NAb) induction

12-day-old female/male rats received daily i.p. administration of vehicle or rapamycin with and without prednisolone starting 2 days before ($t = -2$) until 21 days after ($t = 21$) AAV8-h*UGT1A1* administration (females, 1×10^{12} vg/kg; males, 5×10^{11} vg/kg). At 10 weeks after vector administration the level of anti-AAV8 IgG and NAbs in serum was determined. (A and C) Anti-AAV8 IgG levels in female (A) and male (C) rats. (B and D) Anti-AAV8 NAbs in female (B) and male (D) rats. Positive means positive control for the assay. The neutralizing titer of the sample is determined as the highest dilution at which 50% or greater inhibition of the luciferase expression is measured. Data represent mean ± SD.

irrespective of the presence of prednisolone (Figures 2A and 2B), the efficacy of re-dosing AAV8-h*UGT1A1* was investigated by injecting the animals at P14 with a sub-therapeutic dose of 1×10^{12} vg/kg, and re-injecting the animals at week 14 (W14) with a dose previously shown to provide long-term correction in female rats injected at P28 (5×10^{12} vg/kg, Figure 1B). The rats received an immune-suppressive regimen identical to the first time to investigate whether this could prevent a humoral response in primed animals. In rats receiving the immune suppression the second injection did result in a significant reduction of serum bilirubin, whereas in vehicle-treated rats no effect was seen (Figure 3A; Table 1). This response, however, appeared transient. At 4 weeks after vector re-dosing a 50% reduction of serum bilirubin in rapamycin with and without prednisolone-treated rats was seen, while the effect of re-treatment of initially vehicle-treated rats did not result in a significant reduction of serum total bilirubin. At later time points the difference between both groups did not reach statistical significance, although the levels in rats receiving rapamycin remained below that of the vehicle-treated groups. Excretion of bilirubin conjugates in bile, the most direct parameter of total UGT1A1 activity in liver, and the level of vector genomes in the liver were higher in both rapamycin-treated groups (Figures 3C and 3D). Although these data indicate that rapamycin treatment does improve efficacy of the second treatment, in view of the results in the juvenile rats, this low and transient efficacy was unexpected. To investigate whether this was caused by an immune response not detected by the ELISA and NAb assays, 14-week-old naive rats were treated with 5×10^{12} vg/kg AAV8-h*UGT1A1*. In these rats a transient reduction in serum bilirubin levels, comparable to that seen in both rapamycin groups, was also seen (Figure 3B). In addition, the amount of CB in bile and the presence of vector genomes in these naive animals were also very comparable to those in the re-injected rats (Figures 3C and 3D). Overall, this indicates that the reduced efficacy seems due to age of these female rats at the time of (re)dosing. In males, re-treatment upon loss of correction did result in a persistent therapeutic reduction of serum bilirubin levels (Figure 3E; Table 2). Different from the transient correction in females, at 4 weeks after vector re-dosing a more than 75% reduction of serum bilirubin was found compared to rats receiving a single dose at P14 in males. This increased efficacy is also reflected by the excretion of bilirubin conjugates into bile and vector genomes present in liver at the time of sacrifice (Figures 3F and 3G).

Rapamycin does not prevent a humoral response toward AAV in primed rats

A rapamycin base regimen did prevent the induction of a humoral response in suckling female rats. To investigate whether this treatment would also effectively prevent a response upon re-administration of the vector, all animals received the same immune-suppressive regimen covering the second AAV dosing. NAbs and IgG levels toward AAV8 were determined at week 10 after the second injection. As shown in Figures 4A–4D, immune suppression did not block the induction of NAbs and IgG upon re-injection in both sexes,

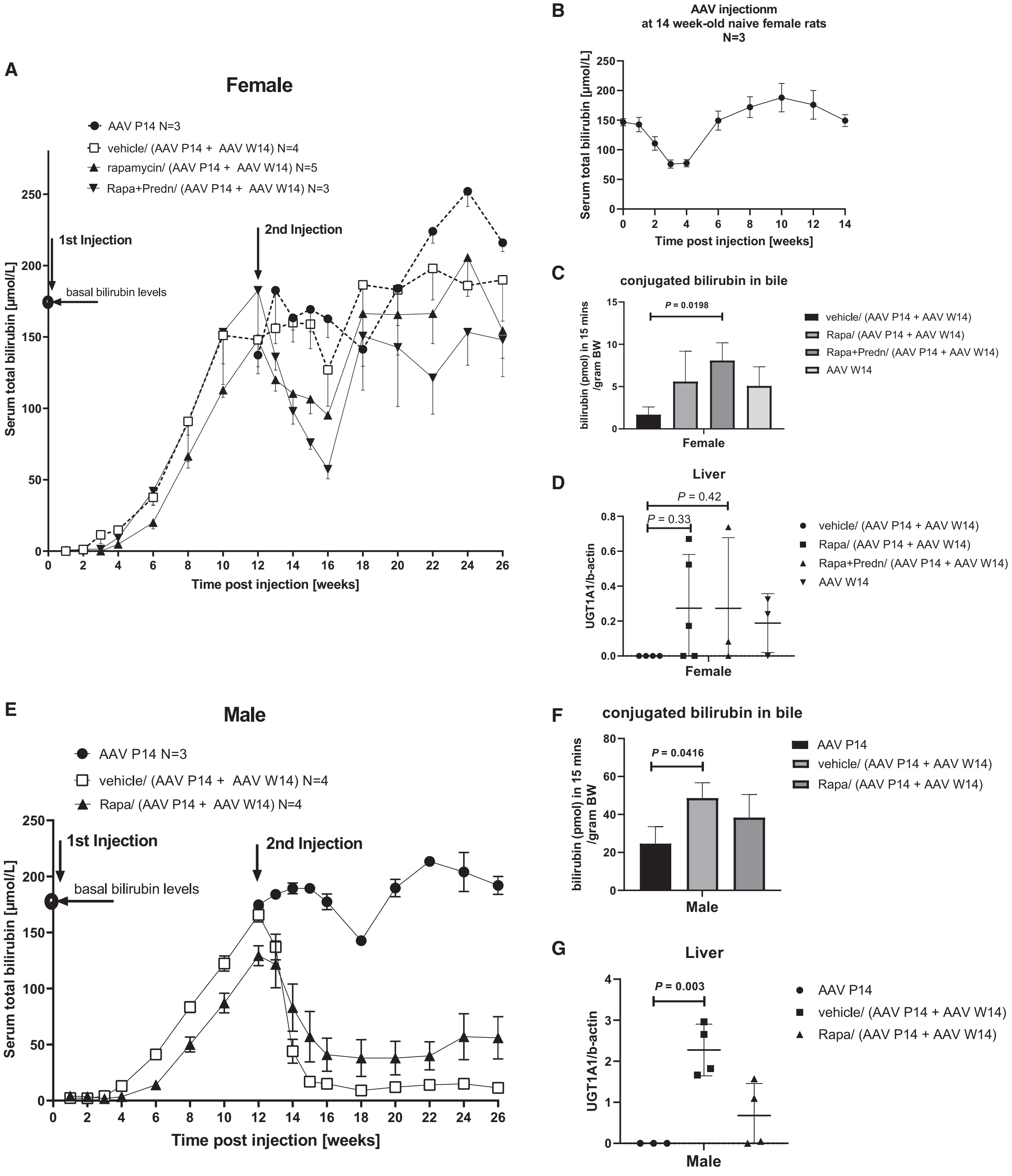

Figure 3. In the absence of NAbs the efficacy of re-administered AAV8-hUGT1A1 is comparable to that in naive rats

At 12 weeks after the first AAV8-h*UGT1A1* administration half of the vehicle-treated rats were re-injected with 5×10^{12} vg/kg of vector. All rats treated the first time with vector in combination with immune suppression were re-dosed with 5×10^{12} vg/kg of AAV8-h*UGT1A1* in combination with the same immune-suppressive regimen. (A and E)

(legend continued on next page)

although it did reduce the titer compared to vehicle-treated rats. In contrast to rats re-injected with AAV, the immune-suppressive regimen did completely block the immune response in the naive rats (Figures S4B–S4E). Also, in the 14-week-old animals the immune-suppressive regimen resulted in a small effect of weight, but the difference did not reach statistical significance (Figure S3) and induced a mild leukopenia (Figure S5).

Rapamycin affects the biodistribution of AAV

To investigate whether the rapamycin treatment modified the biodistribution upon systemic administration of AAV8-h*UGT1A1,* the amount of viral genomes in liver, lung, kidney, and spleen was determined. In all four organs, viral genomes were detectable (Figure S6). In the rats receiving a single injection at P14 the vector genomes were undetectable. In re-injected female rats, in none of the vehicle rats could vector be detected in the liver (Figure 3D). In immune suppression-treated rats and in naive rats, vector genomes were detectable in part of the animals, albeit at a low level. Five- to 10-fold higher levels of vector genomes were present in male livers, but the results were also heterogeneous, with two rapamycin-treated rats having very low levels (Figure 3G). At the time of sacrifice high numbers of vector genomes were detected in the spleen of naive rats injected at week 14 (Figure 5). Presence of vector copies in the spleen and not in the liver could play a role in the loss of efficacy seen in older animals. These increased levels were not seen in the spleen of animals receiving rapamycin. To investigate whether rapamycin could increase the efficacy by preventing vector loss to the spleen, naive 14-week-old males and females were treated with rapamycin as before and received a dose of 5×10^{12} vg/kg AAV8-h*UGT1A1*. Although this indeed reduced the presence of vector genomes in the spleen, it did not improve the treatment efficacy (Figure 5). In males the serum bilirubin levels were reduced with 75%, which is less than that in rats not receiving rapamycin. The 10%–20% reduction in females is comparable to that seen in the retreated animals receiving rapamycin (Figure S4A).

DISCUSSION

Administration of an AAV8-h*UGT1A1* vector is currently being evaluated as a potential treatment of severe unconjugated hyperbilirubinemia, CNs (ClinicalTrials.gov: NCT03466463).[22,23] Although effective phototherapy does allow treatment of adults, the risk for irreversible brain damage in severely affected patients persists and the disease burden during all of these years is significant.[4,5,24] In addition, presence of liver fibrosis was seen in livers from CNs type 1 patients, indicating that this severe syndrome can result in liver damage.[25] Gene therapy at a younger age therefore seems preferable. Furthermore, in view of the gradual increase in the prevalence of preexisting immunity toward AAV vectors during aging, treating at a younger age would be preferable.[13,14] Screening adult CNs type 1 patients showed that 30.6% were not eligible for AAV-mediated gene therapy because of preexisting NAbs toward AAV8.[15]

The episomal persistence of AAV vector genomes, however, compromises long-term efficacy due to dilution of the vector in growing livers. To investigate whether the vector used in the ongoing clinical trial can provide long-term correction at a younger age, a clinically relevant dose was administered to neonatal, suckling, and juvenile rats to model its use in young children suffering from CNs. When treating neonatal rats the correction was lost completely, and only sporadic clusters of UGT1A1-expressing hepatocytes were present, most likely resulting from random integration of the vector in the host genome (Figure 1; Figure S1). Since the vector dose administered (vg/kg) was based on the weight of the animals at the time of treatment, this loss of efficacy can, at least partially, be explained by the lower number of vector genomes given to the neonatal and suckling rats. At the time of sacrifice the presence of vector genomes in the liver was >10-fold lower than that seen with the same vector administered to adult rats.[23] Since the difference in weight between week 4 (P28) and week 8 is less than a factor 2, the difference in absolute vector dose between these two groups is <2-fold. The 10-fold difference of vector in the liver at the time of sacrifice indicates that in addition to vector dilution, hepatocyte proliferation enhances vector loss. A comparable loss of efficacy was reported in this model and in a Ugt1a1 deficient mouse model also reporting presence of some integrations.[11,12,26,27] These data indicate that treatment very early after birth compromises efficacy, with only minimal expression from a few integrated gene copies. Re-treatment using virus serotype switching is an option to overcome the hurdle imposed by the induction of NAbs due to the first treatment, but it is limited by cross-reactivity between serotypes. Moreover, a development process for the production of two different vectors complicates this approach.[27] Gene therapy treatment at P1 limited the induction of NAbs in several rats (data not shown) and in mice allowed effective re-treatment.[26] Treatment very early after birth will be complicated in patients for several reasons. Since physiological jaundice occurs in almost all newborns, and pathological jaundice can be due to several much more frequent causes, jaundice will only be recognized first a few days after delivery and treated with phototherapy. Only upon reappearance of severe jaundice after stopping phototherapy will additional diagnostic tests be performed to diagnose UGT1A1 deficiency and to establish the severity. Only the severe form of CNs should be treated with gene therapy.[2] Thus, treatment at a juvenile age seems more representative to model gene therapy treatment for CN patients. In suckling rats treated at P14, the loss of correction is less compared to P1, although already at 12 weeks after treatment a significant increase in serum bilirubin is seen in females. This more prominent loss in females may be due to the lower efficacy of AAV vectors to transduce females reported for murine animals.[20,21] In contrast to treatment at P1, the

Serum bilirubin levels over time in female (A) and male (E) rats. (B) Serum bilirubin levels over time in 14-week-old naive females upon receiving 5×10^{12} vg/kg of AAV8-h*UGT1A1* vector. (C and F) Presence of conjugated bilirubin in bile of female (C) and male (F) rats. (D and G) Vector genome copies in female (D) and male (G) liver at the time of sacrifice. Data represent the mean ± SD. Statistical analyses were performed by one-way ANOVA with a Tukey post hoc test and for serial measurements of serum bilirubin with a mixed-effects assay.

Table 1. Reduction in serum bilirubin in female rats in different groups

	Serum bilirubin, p value versus AAV P14	
	13–16 weeks	18–26 weeks
AAV P14	170 ± 27 μM	204 ± 43 μM
Vehicle/(AAV P14 + AAV W14)	151 ± 33 μM, p = 0.2281	189 ± 40 μM, p = 0.8458
Rapamycin/(AAV P14 + AAV W14)	108 ± 23 μM, p = 0.0008	172 ± 49 μM, p = 0.3173
Rapamycin/prednisolone/(AAV P14 + AAV W14)	92 ± 33 μM, p = 0.0297	143 ± 48 μM, p = 0.1245

Average serum bilirubin was calculated during periods of 13–16 and 18–26 weeks after injection. Mean ± SD. A mixed effects analysis of variance was used to calculate p values.

Table 2. Reduction in serum bilirubin in male rats in different groups

	Serum bilirubin, p value versus AAV P14	
	13–16 weeks	18–26 weeks
AAV P14	185 ± 9 μM	188 ± 29 μM
Vehicle/(AAV P14 + AAV W14)	53 ± 53 μM, p = 0.0388	12 ± 5 μM, p = 0.0002
Rapamycin/(AAV P14 + AAV W14)	76 ± 48 μM, p = 0.0151	46 ± 31 μM, p = 0.0004

Average serum bilirubin was calculated during periods of 13–16 and 18–26 weeks after injection. Mean ± SD. A mixed effects analysis of variance was used to calculate p values.

intravenous administration of a clinical dose at P14 resulted in the presence of anti-AAV antibodies in all animals, indicating that this is a good time for injection to study the efficacy of immune suppression to allow effective re-administration.

Our results showed that a 3-week course of rapamycin prevents the humoral response against AAV in naive suckling and adult female rats (Figures 2 and 4). In NHPs an immune-suppressive regimen containing rapamycin combined with additional immune suppressants such as mycophenolate mofetil (MMF) was not sufficient to completely block anti-AAV capsid responses.[28,29] Since in that study a higher dose was used and the immune suppression was continued up to 70 days after vector injection, the most likely explanation for the observed difference is exposure of the macaques to an endogenous AAV serotype, prior to vector administration. In our study, rapamycin treatment did not block the humoral response when the vector was re-administered, while it was effective in naive adult rats (Figure S4). Thus, although rapamycin prevented the induction of a detectable humoral response upon the first injection, the animals were primed and not naive toward this vector anymore. This observation will have important implications when aiming to prevent the humoral response toward the AAV capsid in patients. Since AAV is endemic in humans, patients will be exposed to this vector during life, and in most adults the immune system may be primed toward AAV. The presence of some sort of an immune memory toward AAV in adults was demonstrated by the immune-mediated loss of correction seen in patients in the first trial to treat hemophilia B, while in the naive dog model lifelong correction was established.[30] Although this exposure to natural AAV does not always result in detectable antibody levels, it will make rapamycin treatment to prevent the induction of NAbs ineffective probably in most adult patients. In addition, rapamycin did not enhance the correction of serum bilirubin levels, while in NHPs it did enhance liver transduction.[18] The most likely explanation for these contradicting results is the different protocol used. While in the NHP a single administration to enhance autophagy was used, preventing a humoral response against AAV capsids required a prolonged treatment that may cause liver toxicity and increase inflammation.[31] Encapsulating rapamycin in nanoparticles may overcome both problems. A single injection of those nanoparticles appeared to be effective and did allow re-administration of a vector at 30 days after the initial dosing in NHPs.[32] Safety and efficacy studies of this promising strategy are ongoing in Ugt1a1 deficient murine models to investigate whether this approach can be applicable to CNs patients.

This targeted approach may also reduce the adverse effects of a 3-week course of rapamycin. Our data indicate that this had a minor non-significant delay effect in the growth of the suckling rats (Figures S3A and 3B). The growth retardation of a 3-week course in children may be smaller due to a slower growth, as rats double their weight from week 2 to 5 after birth, while in children the increase is <20% during the first month, but this is an unwanted side effect. In this respect a single injection of a targeted rapamycin also seems preferable. Other effects of the rapamycin treatment, such as a mild leukopenia, are expected and the effect is only transient and does not appear to render this approach less feasible.

The transient correction in 14-week-old female rats (Figure 3) using a dose of 5×10^{12} vg/kg is comparable to the correction of serum bilirubin levels previously obtained with a 10-fold lower dose of this vector.[22] As shown in Figure 1, in 4-week-old juvenile rats this dose did provide sustained therapeutic correction. Also, in female rats 6–8 weeks old, this dose proved to be effective.[22,23] This indicates that in the 14-week-old female rats AAV8 liver transduction appears less efficient. In male rats the difference in efficacy between the different ages was much smaller, but when administered at week 14, a low level of bilirubin was detectable while at earlier time points after treatment no bilirubin was detectable in serum (Figures 1 and 3). Others have demonstrated that the higher efficiency in males involves an androgen-dependent pathway enhancing single- to double-strand DNA conversion.[20,21] This androgen-dependent pathway cannot explain the significant lower efficacy in older female rats compared to juvenile females. In 9 of 22 CNs type 1 patients liver fibrosis was seen in a recent study, which could impair AAV liver transduction efficiency.[25] The presence of liver damage in older female rats is however unlikely. First, liver enzymes were in the normal range during the entire study and no liver damage was seen at the time of sacrifice (Figures S7 and S8). In addition, in 2-year-old rats no liver damage was detectable.[33] Furthermore, severe liver damage in a mouse model with cirrhosis did not impair AAV transduction efficiency.[34] In this respect, the increased presence of vector ending up in the spleen could play a role in the

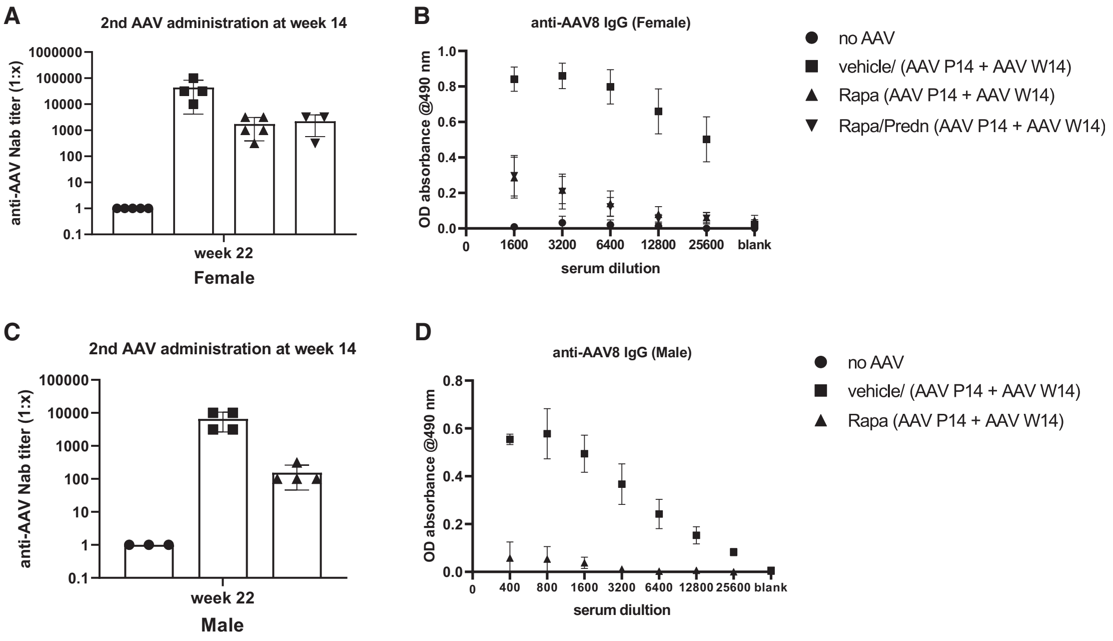

Figure 4. Rapamycin does not prevent humoral response in rats previously exposed to AAV

14-week-old previously injected female/male rats received daily i.p. administration of vehicle or rapamycin with and without prednisolone starting 2 days before (t = −2) until 21 days after (t = 21) AAV8-h*UGT1A1* (re)administration. At 10 weeks after vector administration the level of anti-AAV8 IgG and NAbs in serum was determined. (A and C) Anti-AAV8 NAbs in female (A) and male (C) rats. (B and D) Anti-AAV8 IgG levels in female (B) and male (D) rats. The neutralizing titer of the sample is determined as the highest dilution at which 50% or greater inhibition of the luciferase expression is measured. Data represent the mean ± SD.

reduced efficacy in older animals (Figures 5A and 5B). The presence of NAbs does cause increased uptake of AAV vectors by the spleen in rats and mice.[17,31] Treating the animals with rapamycin for 3 weeks reduced the presence of vector in the spleen, most likely by delaying the induction of NAbs until most of the vector had disappeared from the circulation (Figure S9). This prolonged rapamycin treatment did not prevent the induction of NAbs at later time points and did not improve the correction, indicating that it appears not to be a promising approach to improve AAV efficacy. Several studies have shown macrophages do phagocytose AAV vectors and, as such, are involved in the innate immune response toward this vector.[35,36] Previously we have shown that preventing the uptake of a scAAVLP1-*UGT1A1* by these cells does improve correction of serum bilirubin levels and liver transduction, especially in female Gunn rats.[37] The latter suggests that in addition to the reported less efficient single- to double-stranded conversion of AAV vector genomes in females, the higher number of Kupffer cells and their higher phagocytosis activity may be responsible for the reduced efficacy.[38–40] This sexual dimorphism also exists in humans and may affect the vector efficacies, especially in adults.

Overall, our studies showed that AAV-mediated *in vivo* gene therapy to provide long-term correction of inherited hyperbilirubinemia in Gunn rats is feasible at P28. Earlier treatment results in loss of efficacy. How this translates to human age requires studies comparing liver growth rates in children and juvenile rats. Rapamycin could prevent the induction of NAbs in naive animals. However, its potential effect on growth and its poor effectivity after prior exposure to AAV, which is endemic in the human population, indicates that other treatments are needed to prevent the NAbs. Depletion of AAV NAbs using immune adsorption does allow efficient liver transduction with AAV but seems only sufficiently effective when antibody titers are low.[41] Other options such as removal of IgGs using endopeptidase therefore seem more promising.[42,43]

MATERIALS AND METHODS

Animal study

Ugt1a1 deficient Gunn rats from our own breeding colony received AAV8-h*UGT1A1* (produced by Genethon, France; n = 6–8 of equal sex) treatment by facial vein injection at P1, or by tail vein administration at P14 or P28. Rats were housed in a temperature-controlled environment with a 12-h light/12-h dark cycle and permitted *ad libitum* consumption of C1000 control diet (Altromin, Triple A Trading, Germany) and water. Blood sampling was performed by tail vein puncture under isoflurane anesthesia in heparin tubes. At the time of sacrifice, bile was sampled as previously described,[44] and blood was collected by cardiac puncture and plasma (heparin) was separated by centrifugation at 1,000 × *g* for 5 min. Organs were fixed overnight in 4% paraformaldehyde or snap-frozen in liquid N_2 and stored at −80°C for further analysis. All animal experiments were performed in accordance with the European Directive 2010/63/EU and with

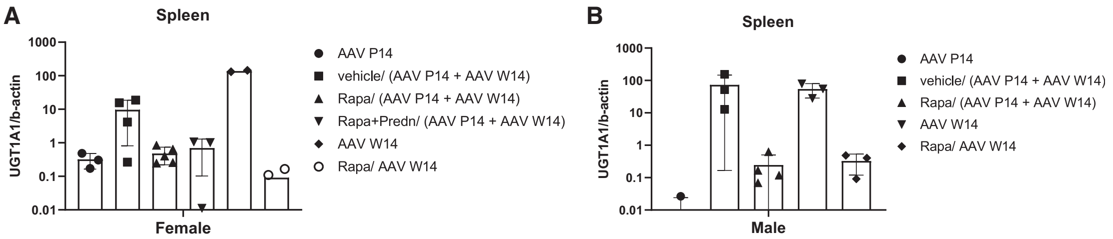

Figure 5. Rapamycin reduces presence of AAV vector genomes in the spleen at the time of sacrifice
(A and B) Vector genome copies in the spleen of female (A) and male (B) rats at the time of sacrifice. Data are shown as mean ± SD.

approval of the Institutional Animal Care and Use Committee of the Amsterdam UMC.

Rapamycin (Sanbio; 15 mg/mL) and prednisolone (Sigma-Aldrich; 50 mg/mL) stock solutions were made in DMSO and stored at −20°C. Stocks were diluted 10 times in 5% Tween 80 (Sigma-Aldrich) and 5% polyethylene glycol 400 (PEG 400) (Hampton Research), as previously described.[45] Rats received 1.5 mg/kg rapamycin or 1.5 mg/kg rapamycin + 5 mg/kg prednisolone or vehicle + DMSO control by a daily i.p. injection, starting 2 days prior to vector administration and ending 21 days after.

Bilirubin quantification

Bilirubin metabolites in bile were quantified by high-performance liquid chromatography (HPLC) as described[44] with the modification that a Pursuit column (Agilent Technologies, the Netherlands) was used. Bilirubin in serum was quantified on a Roche Cobas c502/702 analyzer (Roche Diagnostics, USA) by the hospital Routine Clinical Chemistry Department.

Determination of AAV8 NAbs

The AAV8 NAb titer in plasma was determined using the protocol reported previously.[19] Briefly, plasma samples were preincubated at 56°C for 30 min to inactivate complement. AAV8-luciferase was preincubated for 1 h at 37°C with plasma samples diluted in fetal calf serum (FCS) and added to HEK293T cells cultured at 37°C, 5% CO_2. One day later luciferase expression was determined using the ONE-Glo luciferase assay system (Promega), according to the manufacturer's protocol.

ELISA for AAV8 and h*UGT1A1* antibody

An indirect ELISA approach was used to detect the anti-AAV8 or anti-UGT1A1 IgG in rat serum as previously described.[46] An ELISA plate was coated overnight (O/N) at 4°C with 50 μL/well of lysate from HEK293T cells, HEK293T cells expressing AAV8 capsid, or 1 μg/mL human rUGT1A1 protein (Bio Connect) in 50 mM carbonate coating buffer. The next day the wells were incubated with 200 μL of blocking buffer (1% gelatin in phosphate-buffered saline [PBS]). After 2 h, the blocking buffer was removed and 50 μL/well of heparin plasma samples diluted in washing buffer with 0.05% Tween 20 in PBS was added and incubated for 1.5 hours at room temperature. After three washings with washing buffer pDP8/UGT1A1, binding rat immunoglobulins were detected with horseradish peroxidase (HRP) conjugated anti-rat IgG at 1:1,000 dilution in conjugation buffer containing four-fifths blocking buffer and one-fifth washing buffer followed by *o*-phenylenediamine (Sigma) conversion.

Biodistribution and h*UGT1A1* mRNA levels

Genomic DNA was isolated as previously described[47] from the tissues. h*UGT1A1* copy number was determined by qPCR in a Bio-Rad CFX96 system (Roche Diagnostics, USA) using the TaqMan assay, iQ Supermix (Bio-Rad, Germany), and the primers mentioned in Table S1. Results were processed and analyzed using LinRegPCR software and normalized to β-actin. qRT-PCR was performed on a Bio-Rad CFX96 system (Roche Diagnostics, USA) using the SensiFAST SYBR No-ROX kit (Bioline) and the primers listed (Table S1). Data were normalized to β-actin.

Histology

For conventional bright-field light microscopy, tissues were fixed in 4% formaldehyde solution and embedded in paraffin. Hematoxylin (Sigma, 51275) and eosin (Sigma, E4382) (H&E) staining was performed on tissue sections as previously described.[48]

RNA *in situ* hybridization

Detection of UGT1A1 mRNA in liver tissue was done using RNAscope technology according to the manufacturer's protocol (Advanced Cell Diagnostics, Hayward, CA, USA). Target probes for h*UGT1A1*, based on the sequences listed in Table S1, were hybridized on liver tissue and visualized after signal amplification with diaminobenzidine (DAB), and counterstained using hematoxylin. UBC and DapB probes served as positive and negative controls, respectively.

Statistical analysis

Serum bilirubin data are presented as mean values ± standard error of the mean (SEM) and were analyzed for significance using a mixed-effects analysis. Other data are presented as mean values ± standard deviation (SD) and were analyzed for significance using the independent t test for the comparison of parametric variables between two groups, unless stated otherwise. For nonparametric variables, we performed a Mann-Whitney test, and for the comparison of three or more groups a one-way analysis of variance (ANOVA) was performed using GraphPad Prism 8 software (GraphPad, CA, USA). *$p < 0.05$, **$p < 0.01$, and ***$p < .001$ were considered significant.

SUPPLEMENTAL INFORMATION

Supplemental Information can be found online at https://doi.org/10.1016/j.omtm.2020.11.016.

ACKNOWLEDGMENTS

This work was supported by a grant from ZonMw, the Netherlands (Translational Gene Therapy Research TGO 43300003 to P.J.B.), and CureCN has received funding from the European Union's Horizon 2020 Research and Innovation Program under grant agreement no. 755225. X.S. is recipient of a fellowship from the China Scholarship Council (CSC).

AUTHOR CONTRIBUTIONS

Study Design, X.S., and P.J.B.; Data Collection, Analysis, and Interpretation, X.S., S.J.A., L.t.B., S.D., R.S.B., and D.R.d.W; Manuscript Preparation, X.S., and P.J.B.; Critical Appraisal and Manuscript Review and Editing, P.J.B., G.R., A.F.M., F.M., G.B., F.C., and R.P.O.E.

DECLARATION OF INTERESTS

F.C., G.B., G.R., A.F.M., and F.M. are inventors of patents describing liver gene transfer approaches for metabolic diseases and/or treatment of hyperbilirubinemia. F.M. is an employee of Spark Therapeutics, a Roche company. The remaining authors declare no competing interests.

REFERENCES

1. Crigler, J.F., Jr., and Najjar, V.A. (1952). Congenital familial nonhemolytic jaundice with kernicterus. Pediatrics *10*, 169–180.
2. Bosma, P.J. (2003). Inherited disorders of bilirubin metabolism. J. Hepatol. *38*, 107–117.
3. Erlinger, S., Arias, I.M., and Dhumeaux, D. (2014). Inherited disorders of bilirubin transport and conjugation: new insights into molecular mechanisms and consequences. Gastroenterology *146*, 1625–1638.
4. Strauss, K.A., Robinson, D.L., Vreman, H.J., Puffenberger, E.G., Hart, G., and Morton, D.H. (2006). Management of hyperbilirubinemia and prevention of kernicterus in 20 patients with Crigler-Najjar disease. Eur. J. Pediatr. *165*, 306–319.
5. van der Veere, C.N., Sinaasappel, M., McDonagh, A.F., Rosenthal, P., Labrune, P., Odièvre, M., Fevery, J., Otte, J.B., McClean, P., Bürk, G., et al. (1996). Current therapy for Crigler-Najjar syndrome type 1: report of a world registry. Hepatology *24*, 311–315.
6. Tu, Z.H., Shang, D.S., Jiang, J.C., Zhang, W., Zhang, M., Wang, W.L., Lou, H.Y., and Zheng, S.S. (2012). Liver transplantation in Crigler-Najjar syndrome type I disease. Hepatobiliary Pancreat. Dis. Int. *11*, 545–548.
7. Adam, R., Karam, V., Delvart, V., O'Grady, J., Mirza, D., Klempnauer, J., Castaing, D., Neuhaus, P., Jamieson, N., Salizzoni, M., et al.; All contributing centers (www.eltr.org); European Liver and Intestine Transplant Association (ELITA) (2012). Evolution of indications and results of liver transplantation in Europe. A report from the European Liver Transplant Registry (ELTR). J. Hepatol. *57*, 675–688.
8. George, L.A., Sullivan, S.K., Giermasz, A., Rasko, J.E.J., Samelson-Jones, B.J., Ducore, J., Cuker, A., Sullivan, L.M., Majumdar, S., Teitel, J., et al. (2017). Hemophilia B gene therapy with a high-specific-activity factor IX variant. N. Engl. J. Med. *377*, 2215–2227.
9. Nathwani, A.C., Reiss, U.M., Tuddenham, E.G., Rosales, C., Chowdary, P., McIntosh, J., Della Peruta, M., Lheriteau, E., Patel, N., Raj, D., et al. (2014). Long-term safety and efficacy of factor IX gene therapy in hemophilia B. N. Engl. J. Med. *371*, 1994–2004.
10. Colella, P., Ronzitti, G., and Mingozzi, F. (2017). Emerging issues in AAV-mediated *in vivo* gene therapy. Mol. Ther. Methods Clin. Dev. *8*, 87–104.
11. Bortolussi, G., Zentillin, L., Vaníkova, J., Bockor, L., Bellarosa, C., Mancarella, A., Vianello, E., Tiribelli, C., Giacca, M., Vitek, L., and Muro, A.F. (2014). Life-long correction of hyperbilirubinemia with a neonatal liver-specific AAV-mediated gene transfer in a lethal mouse model of Crigler-Najjar syndrome. Hum. Gene Ther. *25*, 844–855.
12. Flageul, M., Aubert, D., Pichard, V., Nguyen, T.H., Nowrouzi, A., Schmidt, M., and Ferry, N. (2009). Transient expression of genes delivered to newborn rat liver using recombinant adeno-associated virus 2/8 vectors. J. Gene Med. *11*, 689–696.
13. Mimuro, J., Mizukami, H., Shima, M., Matsushita, T., Taki, M., Muto, S., Higasa, S., Sakai, M., Ohmori, T., Madoiwa, S., et al. (2014). The prevalence of neutralizing antibodies against adeno-associated virus capsids is reduced in young Japanese individuals. J. Med. Virol. *86*, 1990–1997.
14. Perocheau, D.P., Cunningham, S., Lee, J., Antinao Diaz, J., Waddington, S.N., Gilmour, K., Eaglestone, S., Lisowski, L., Thrasher, A.J., Alexander, I.E., et al. (2019). Age-related seroprevalence of antibodies against AAV-LK03 in a UK population cohort. Hum. Gene Ther. *30*, 79–87.
15. Aronson, S.J., Veron, P., Collaud, F., Hubert, A., Delahais, V., Honnet, G., de Knegt, R.J., Junge, N., Baumann, U., Di Giorgio, A., et al. (2019). Prevalence and relevance of pre-existing anti-adeno-associated virus immunity in the context of gene therapy for Crigler-Najjar syndrome. Hum. Gene Ther. *30*, 1297–1305.
16. Biswas, M., Sarkar, D., Kumar, S.R., Nayak, S., Rogers, G.L., Markusic, D.M., Liao, G., Terhorst, C., and Herzog, R.W. (2015). Synergy between rapamycin and FLT3 ligand enhances plasmacytoid dendritic cell-dependent induction of $CD4^+CD25^+FoxP3^+$ Treg. Blood *125*, 2937–2947.
17. Fitzpatrick, Z., Leborgne, C., Barbon, E., Masat, E., Ronzitti, G., van Wittenberghe, L., Vignaud, A., Collaud, F., Charles, S., Simon Sola, M., et al. (2018). Influence of pre-existing anti-capsid neutralizing and binding antibodies on AAV vector transduction. Mol. Ther. Methods Clin. Dev. *9*, 119–129.
18. Hösel, M., Huber, A., Bohlen, S., Lucifora, J., Ronzitti, G., Puzzo, F., Boisgerault, F., Hacker, U.T., Kwanten, W.J., Klöting, N., et al. (2017). Autophagy determines efficiency of liver-directed gene therapy with adeno-associated viral vectors. Hepatology *66*, 252–265.
19. Meliani, A., Leborgne, C., Triffault, S., Jeanson-Leh, L., Veron, P., and Mingozzi, F. (2015). Determination of anti-adeno-associated virus vector neutralizing antibody titer with an in vitro reporter system. Hum. Gene Ther. Methods *26*, 45–53.
20. Davidoff, A.M., Ng, C.Y., Zhou, J., Spence, Y., and Nathwani, A.C. (2003). Sex significantly influences transduction of murine liver by recombinant adeno-associated viral vectors through an androgen-dependent pathway. Blood *102*, 480–488.
21. Pañeda, A., Vanrell, L., Mauleon, I., Crettaz, J.S., Berraondo, P., Timmermans, E.J., Beattie, S.G., Twisk, J., van Deventer, S., Prieto, J., et al. (2009). Effect of adeno-associated virus serotype and genomic structure on liver transduction and biodistribution in mice of both genders. Hum. Gene Ther. *20*, 908–917.
22. Ronzitti, G., Bortolussi, G., van Dijk, R., Collaud, F., Charles, S., Leborgne, C., Vidal, P., Martin, S., Gjata, B., Sola, M.S., et al. (2016). A translationally optimized AAV-UGT1A1 vector drives safe and long-lasting correction of Crigler-Najjar syndrome. Mol. Ther. Methods Clin. Dev. *3*, 16049.
23. Collaud, F., Bortolussi, G., Guianvarc'h, L., Aronson, S.J., Bordet, T., Veron, P., Charles, S., Vidal, P., Sola, M.S., Rundwasser, S., et al. (2018). Preclinical development of an AAV8-hUGT1A1 vector for the treatment of Crigler-Najjar syndrome. Mol. Ther. Methods Clin. Dev. *12*, 157–174.
24. Dhawan, A., Lawlor, M.W., Mazariegos, G.V., McKiernan, P., Squires, J.E., Strauss, K.A., Gupta, D., James, E., and Prasad, S. (2020). Disease burden of Crigler-Najjar syndrome: systematic review and future perspectives. J. Gastroenterol. Hepatol. *35*, 530–543.
25. Mitchell, E., Ranganathan, S., McKiernan, P., Squires, R.H., Strauss, K., Soltys, K., Mazariegos, G., and Squires, J.E. (2018). Hepatic parenchymal injury in Crigler-Najjar type I. J. Pediatr. Gastroenterol. Nutr. *66*, 588–594.
26. Greig, J.A., Nordin, J.M.L., Draper, C., Bell, P., and Wilson, J.M. (2018). AAV8 gene therapy rescues the newborn phenotype of a mouse model of Crigler-Najjar. Hum. Gene Ther. *29*, 763–770.
27. Bočkor, L., Bortolussi, G., Iaconcig, A., Chiaruttini, G., Tiribelli, C., Giacca, M., Benvenuti, F., Zentilin, L., and Muro, A.F. (2017). Repeated AAV-mediated gene

transfer by serotype switching enables long-lasting therapeutic levels of hUgt1a1 enzyme in a mouse model of Crigler-Najjar syndrome type I. Gene Ther. *24*, 649–660.

28. Mingozzi, F., Hasbrouck, N.C., Basner-Tschakarjan, E., Edmonson, S.A., Hui, D.J., Sabatino, D.E., Zhou, S., Wright, J.F., Jiang, H., Pierce, G.F., et al. (2007). Modulation of tolerance to the transgene product in a nonhuman primate model of AAV-mediated gene transfer to liver. Blood *110*, 2334–2341.

29. Jiang, H., Couto, L.B., Patarroyo-White, S., Liu, T., Nagy, D., Vargas, J.A., Zhou, S., Scallan, C.D., Sommer, J., Vijay, S., et al. (2006). Effects of transient immunosuppression on adenoassociated, virus-mediated, liver-directed gene transfer in rhesus macaques and implications for human gene therapy. Blood *108*, 3321–3328.

30. Manno, C.S., Pierce, G.F., Arruda, V.R., Glader, B., Ragni, M., Rasko, J.J., Ozelo, M.C., Hoots, K., Blatt, P., Konkle, B., et al. (2006). Successful transduction of liver in hemophilia by AAV-factor IX and limitations imposed by the host immune response. Nat. Med. *12*, 342–347.

31. Umemura, A., Park, E.J., Taniguchi, K., Lee, J.H., Shalapour, S., Valasek, M.A., Aghajan, M., Nakagawa, H., Seki, E., Hall, M.N., and Karin, M. (2014). Liver damage, inflammation, and enhanced tumorigenesis after persistent mTORC1 inhibition. Cell Metab. *20*, 133–144.

32. Meliani, A., Boisgerault, F., Hardet, R., Marmier, S., Collaud, F., Ronzitti, G., Leborgne, C., Costa Verdera, H., Simon Sola, M., Charles, S., et al. (2018). Antigen-selective modulation of AAV immunogenicity with tolerogenic rapamycin nanoparticles enables successful vector re-administration. Nat. Commun. *9*, 4098.

33. Seppen, J., Bakker, C., de Jong, B., Kunne, C., van den Oever, K., Vandenberghe, K., de Waart, R., Twisk, J., and Bosma, P. (2006). Adeno-associated virus vector serotypes mediate sustained correction of bilirubin UDP glucuronosyltransferase deficiency in rats. Mol. Ther. *13*, 1085–1092.

34. Sobrevals, L., Enguita, M., Rodriguez, C., Gonzalez-Rojas, J., Alzaguren, P., Razquin, N., Prieto, J., and Fortes, P. (2012). AAV vectors transduce hepatocytes in vivo as efficiently in cirrhotic as in healthy rat livers. Gene Ther. *19*, 411–417.

35. Zaiss, A.K., Liu, Q., Bowen, G.P., Wong, N.C., Bartlett, J.S., and Muruve, D.A. (2002). Differential activation of innate immune responses by adenovirus and adeno-associated virus vectors. J. Virol. *76*, 4580–4590.

36. Zaiss, A.K., Cotter, M.J., White, L.R., Clark, S.A., Wong, N.C., Holers, V.M., Bartlett, J.S., and Muruve, D.A. (2008). Complement is an essential component of the immune response to adeno-associated virus vectors. J. Virol. *82*, 2727–2740.

37. van Dijk, R., Montenegro-Miranda, P.S., Riviere, C., Schilderink, R., ten Bloemendaal, L., van Gorp, J., Duijst, S., de Waart, D.R., Beuers, U., Haisma, H.J., and Bosma, P.J. (2013). Polyinosinic acid blocks adeno-associated virus macrophage endocytosis in vitro and enhances adeno-associated virus liver-directed gene therapy in vivo. Hum. Gene Ther. *24*, 807–813.

38. Jaillon, S., Berthenet, K., and Garlanda, C. (2019). Sexual dimorphism in innate immunity. Clin. Rev. Allergy Immunol. *56*, 308–321.

39. Csaba, G., and Pállinger, E. (2009). Gender dependence in the hormone content of the immune cells. Acta Physiol. Hung. *96*, 45–50.

40. Melgert, B.N., Oriss, T.B., Qi, Z., Dixon-McCarthy, B., Geerlings, M., Hylkema, M.N., and Ray, A. (2010). Macrophages: regulators of sex differences in asthma? Am. J. Respir. Cell Mol. Biol. *42*, 595–603.

41. Salas, D., Kwikkers, K.L., Zabaleta, N., Bazo, A., Petry, H., van Deventer, S.J., Aseguinolaza, G.G., and Ferreira, V. (2019). Immunoadsorption enables successful rAAV5-mediated repeated hepatic gene delivery in nonhuman primates. Blood Adv. *3*, 2632–2641.

42. Monteilhet, V., Saheb, S., Boutin, S., Leborgne, C., Veron, P., Montus, M.F., Moullier, P., Benveniste, O., and Masurier, C. (2011). A 10 patient case report on the impact of plasmapheresis upon neutralizing factors against adeno-associated virus (AAV) types 1, 2, 6, and 8. Mol. Ther. *19*, 2084–2091.

43. Leborgne, C., Barbon, E., Alexander, J.M., Hanby, H., Delignat, S., Cohen, D.M., Collaud, F., Muraleetharan, S., Lupo, D., Silverberg, J., et al. (2020). IgG-cleaving endopeptidase enables in vivo gene therapy in the presence of anti-AAV neutralizing antibodies. Nat. Med. *26*, 1096–1101.

44. Seppen, J., Tada, K., Ottenhoff, R., Sengupta, K., Chowdhury, N.R., Chowdhury, J.R., Bosma, P.J., and Oude Elferink, R.P. (1997). Transplantation of Gunn rats with autologous fibroblasts expressing bilirubin UDP-glucuronosyltransferase: correction of genetic deficiency and tumor formation. Hum. Gene Ther. *8*, 27–36.

45. Chen, C., Liu, Y., Liu, Y., and Zheng, P. (2009). mTOR regulation and therapeutic rejuvenation of aging hematopoietic stem cells. Sci. Signal. *2*, ra75.

46. Seppen, J., van Til, N.P., van der Rijt, R., Hiralall, J.K., Kunne, C., and Elferink, R.P. (2006). Immune response to lentiviral bilirubin UDP-glucuronosyltransferase gene transfer in fetal and neonatal rats. Gene Ther. *13*, 672–677.

47. Montenegro-Miranda, P.S., Pichard, V., Aubert, D., Ten Bloemendaal, L., Duijst, S., de Waart, D.R., Ferry, N., and Bosma, P.J. (2014). In the rat liver, adenoviral gene transfer efficiency is comparable to AAV. Gene Ther. *21*, 168–174.

48. James, J., Bosch, K.S., Aronson, D.C., and Houtkooper, J.M. (1990). Sirius red histophotometry and spectrophotometry of sections in the assessment of the collagen content of liver tissue and its application in growing rat liver. Liver *10*, 1–5.

OMTM, Volume *20*

Supplemental Information

Efficacy of AAV8-h*UGT1A1* with Rapamycin in neonatal, suckling, and juvenile rats to model treatment in pediatric CNs patients

Xiaoxia Shi, Sem J. Aronson, Lysbeth ten Bloemendaal, Suzanne Duijst, Robert S. Bakker, Dirk R. de Waart, Giulia Bortolussi, Fanny Collaud, Ronald P. Oude Elferink, Andrés F. Muro, Federico Mingozzi, Giuseppe Ronzitti, and Piter J. Bosma

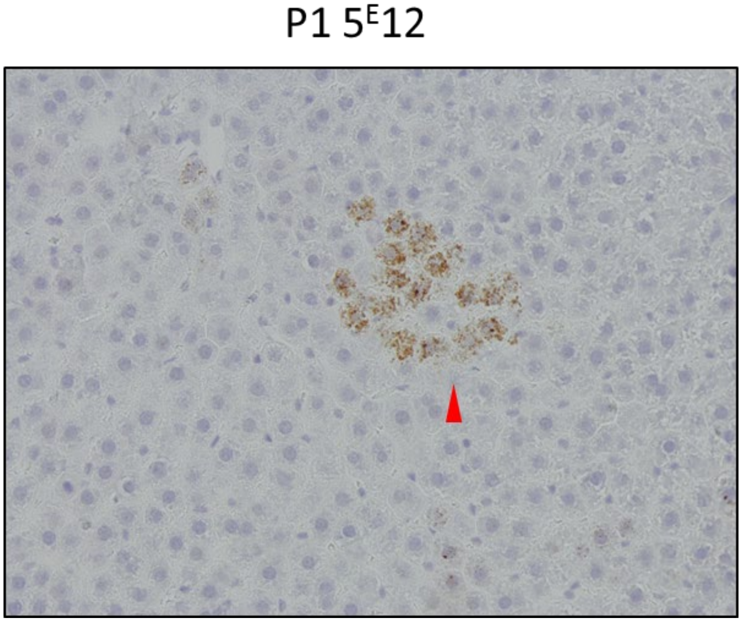

Supplemental Figure 1. Cluster of h*UGT1A* mRNA expressing hepatocytes at 12 weeks after injection of $5x10^{12}$ vg/kg in 1 day old rats.
RNA Scope images (20x) visualizing h*UGT1A* mRNA expression in liver tissue.

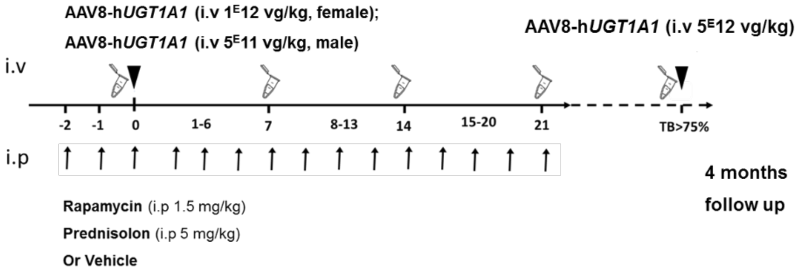

Supplemental Figure 2. Rapamycin experiment setup.
12 days old Gunn rats received daily immune suppressive regimen (IS regimen) by i.p. administration of vehicle, rapamycin with or without prednisolone. Two days later, a suboptimal dose of AAV8-h*UGT1A1* was administered by tail vein injection. The IS regimen continued up to 21 days after vector administration. Upon loss of correction, serum total bilirubin level > 75% of untreated rats, AAV8-*hUGT1A1* (5x10e12 vg/kg) was re-administered in combination with the same IS regimen. Blood was sampled by tail vein puncture to monitor serum total bilirubin, and to determine (neutralizing) antibodies towards AAV8 capsid.

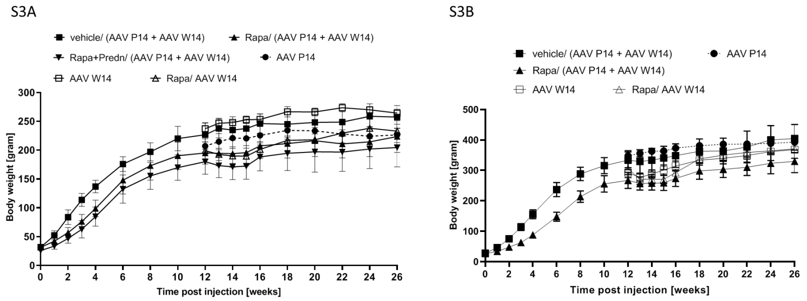

Supplemental Figure 3. Rapamycin with or without prednisolone delay the growth of rats.
The body weight of female (A) and male (B) rats were measured every 1 or 2 weeks after AAV administration. Rats in each group n=3-5. Data are shown as mean ± SD.

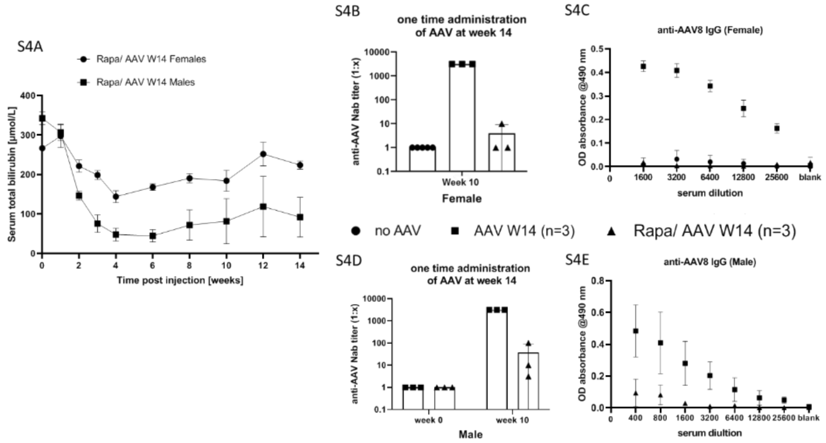

Supplemental Figure 4. Rapamycin prevents the humoral immune response against AAV8 in 14 week old rats, but does not increase liver transduction efficiency.
14 weeks old naïve rats received i.v. administration of 5 x 1012 vg/kg AAV8-hUGT1A1 (n=3) in combination with i.p. administration of daily i.p. injections of rapamycin, starting 2 days before vector until 21 days after vector administration. Blood was sampled by tail vein puncture every 1 or 2 weeks to monitor serum total bilirubin (A). At 10 weeks after AAV administration the level of anti-AAV8 IgG and neutralizing antibodies in serum was determined. Anti-AAV8 neutralizing antibodies (NAbs) in female (B) and male (D). Anti-AAV8 IgG levels in female (C), male (E) rats. Data are shown as mean ± SD.

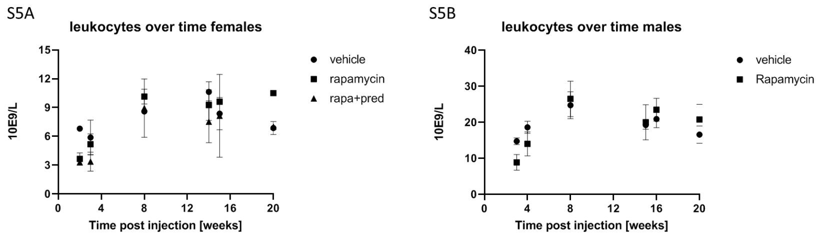

Supplemental Figure 5. Rapamycin and prednisolone result in mild leukopenia in rats.
The leukocytes levels in rats receiving i.p administration of vehicle, rapamycin with or without prednisolone at week 1-3 and week 12-15 were determined at the after the last i.p. injection. (A) and male (B). Rats in each group n=3-5. Data are shown as mean ± SD.

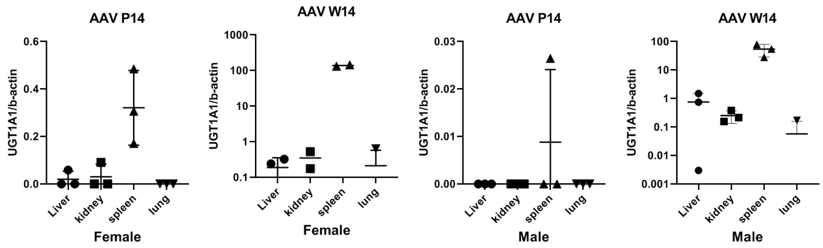

Supplemental Figure 6. Increased presence of AAV vector genomes in the spleen in older animals.
The vector genomic copies in tissues of rats that were naïve at the time of AAV administration that were injected only once, at P14 or W14, and did not receive any immune suppressive. Data are shown as mean ± SD.

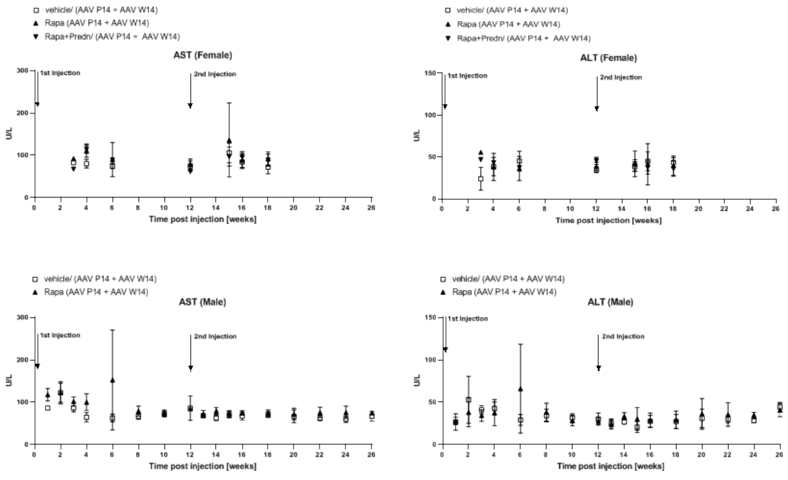

Supplemental Figure 7. Levels of serum AST and ALT overtime.
Blood was sampled by tail vein puncture every 1-2 weeks after AAV8-h*UGT1A1* administration and aspartate aminotransferase (AST) and alanine aminotransferase (ALT) in plasma were determined. The data presented are the mean ± SD at different time points for all groups.

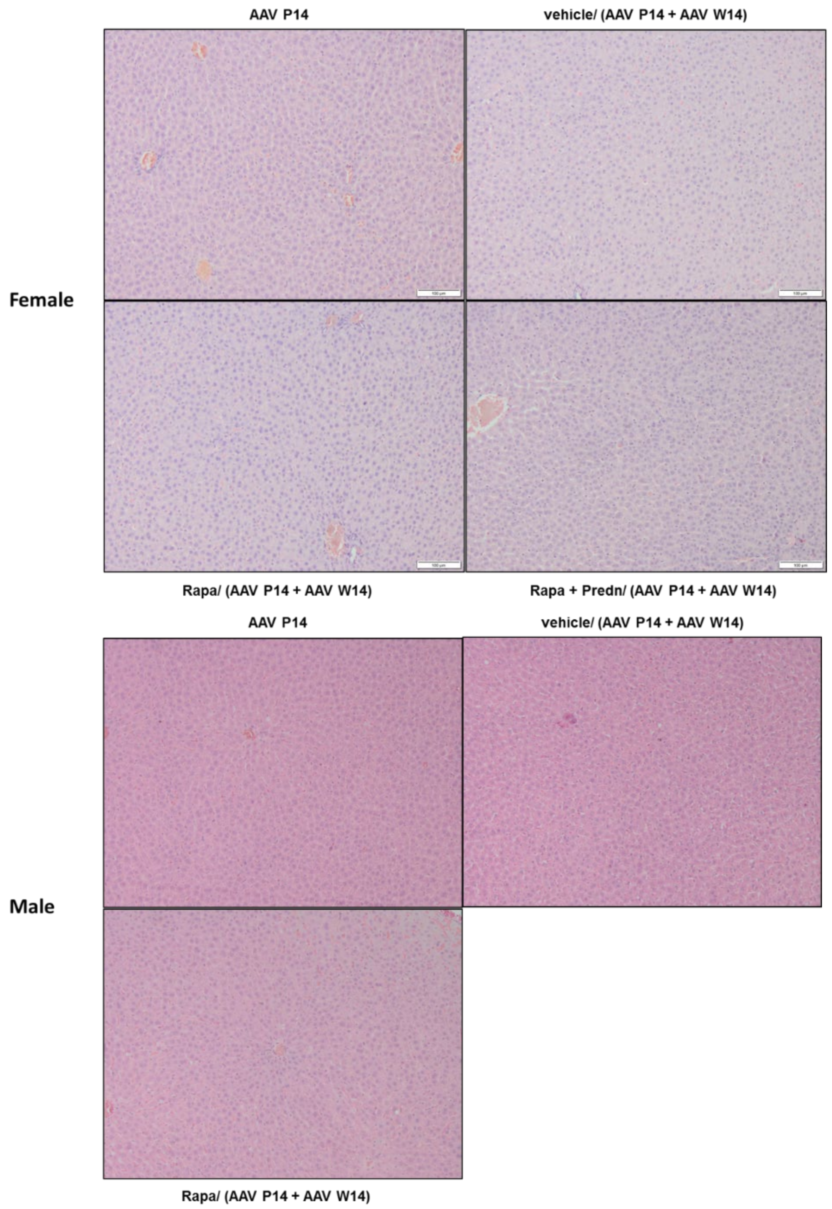

Supplemental Figure 8. Representative images of liver HE staining of rats (20x) at the time of sacrifice.

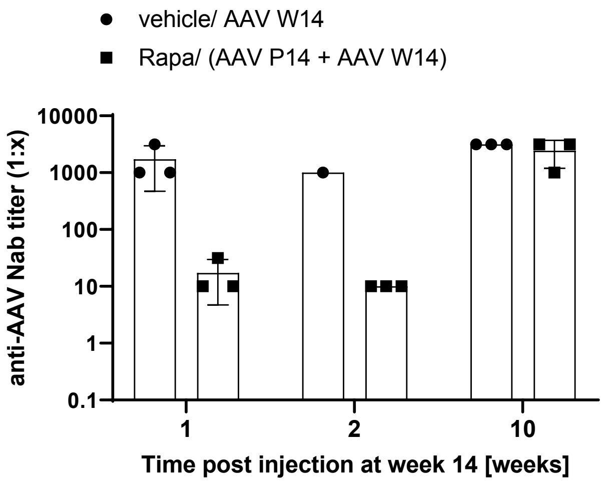

Supplemental Figure 9. Rapamycin treatment delays but does not prevent AAV NAbs formation after the second administration of vector.
Previously injected female rats, received daily i.p. administration of rapamycin starting 2 days before (t = -2) until 21 days (t=21) after re-administration of AAV8-h*UGT1A1* in 14 weeks old animals. 14 weeks old naïve rats received the same dose of AAV8-h*UGT1A1*. At 1, and 2 weeks and 10 weeks after vector administration the level of neutralizing antibodies in serum was determined. Data represent the mean ± SD.

Supplemental Table 1.

Oligonucleotide qRT-PCR primers used for vector detection of *hUGT1A1* in gDNA and cDNA isolated from liver, as well as probes for RNA *in situ* hybridization on liver tissue. And the reference gene *β-actin*.

Target	Sense	Antisense
h*UGT1A1*	5′-GGCGGGCGACTCAGATC-3′	5′-GGGAGGCTGCTGGTGAATATT-3′
β-actin	5′-AGCCATGTACGTAGCCATCCA-3′	5′-TCTCCGGAGTCCATCACAATG-3′
